# Editorial: Housing and Health: Finding Innovative Solutions to Improve the Quality and Condition of the Home

**DOI:** 10.3389/fpubh.2018.00386

**Published:** 2019-01-15

**Authors:** Alesia Ferguson

**Affiliations:** Built Environment Department, North Carolina Agricultural and Technical State University, Greensboro, NC, United States

**Keywords:** housing and health, healthy homes, innovative strateogies for reducing housing hazards, federal enactment of housing standard, home hazards

How important is having a healthy home for individuals, families, and communities across world? One must question the understanding of the concept of a healthy home and also the understanding of its importance to health. The concept of a healthy home may not be shared from individual to individual or from leader to leader within a country or community, and may be driven by historical and economic factors, along with current and demanding needs/drivers within a community.

There are some obvious factors that affect achieving a healthy home that we can all easily understand. Aging homes, for example, present more problems as ventilations systems fail and structural damage grows. Location of a home also plays a part, where for example, more humid and hot climates create more of a challenge in managing pests that frequent homes and threaten health (e.g., cockroaches, rats). The condition of the home can be transient due to external factors. For example, it is devastating to communities and individuals when homes are destroyed due to extreme flooding and wind events from storms. The economics of an area can change, especially in the short term making it difficult to maintain healthy homes to some reasonable standard. We can also see how the quality and standard of a home is affected by the wealth and knowledge of an individual and the community. There are steps that homeowners can take to promote a healthy home where some require few resources (e.g., clean, unclogging drains, keeping shrubs low), while others steps require substantial financial investment (e.g., properly sized and efficient heating and cooling system, roof repairs). Homeowners on various economic scales must be motivated however to see the benefit of various inexpensive and expensive steps as valuable to comfort and health. Renters are also additionally challenged by modifications and repairs that are the Landlord's responsibility.

Communities may also question how much of a role governments play in encouraging healthy homes by driving the adoption of and compliance with updated building, sanitation, and renovation codes, and in providing assistance to those in need.

This research topic received four article submissions. “Community mobilization for slum upgrading through sanitation in Roma informal settlements in the Paris region” by author Chaudhuri explored the social, economic, and political drivers that influence the availability and condition of housing and ultimately its health effects for community members of this informal settlement in Europe. We are reminded by reading this article of desperate conditions of marginalized groups even in wealthy communities, where basic sanitation (i.e., availability of a proper functioning toilet and hot water) can lead to immediate health outcomes such as infections and various levels of discomfort.

Bachelder et al. present results of a survey delivered to over 1,000 renters in a State without an explicit landlord implied warranty of habitability. Over one quarter of the tenants self-report health problems due to substandard housing. Horwitz-Wills offers a perspective on this article by encouraging leaders to include Landlords in state public health systems in order to improve the Landlord-Tenant relationship and renter health. There is again a recognition of the challenges brought on by sociopolitical factors in a community where marginalized renters are challenged to not only find, but to maintain a healthy home.

Ferguson and Yates offer a solution to substandard housing here in the United States which can be duplicated elsewhere and that is a “Federal Enactment of Healthy Homes Legislation in the United States to Improve Health” forcing local governments to put some effort forth in ensuring healthy homes in their communities. Governments must invest in basic standards for homes for individuals and families tailored to meet geographic and local challenges that may impact housing quality. These standards must go beyond a roof and a bed and consider the elements that lead to discomfort, respiratory, and dermal diseases and accidents in and around the home (i.e., proper ventilation, smoke and carbon monoxide detectors, avoidance of trip/fall hazards). The community as a whole pays the price in health care cost for poor and inadequate housing for its most in-need members.

In conclusion, the papers on this research topic explored only a few concepts having to do with healthy homes and innovative solutions. In the future we hope to obtain more articles on this topic exploring strategies to impact homes and achieve some basic and reasonable standard for a broader set of individuals. There are strategies explored and yet to be explored that can take advantage of community, educational, technological, and engineering approaches to reducing biological, chemical, physical, and emotional hazards in housing. The transient nature of housing and home due to family changing economics and natural and manmade disasters also calls on more advanced innovative strategies that will build more permanent and structural sound housing to last and withstand the elements in a cost-effective manner. Climate change (i.e., extremes of hot and cold and more damaging storms) will challenge how we interact with the earth and how we build to shelter more efficiently from the elements.

## Author Contributions

The author confirms being the sole contributor of this work and has approved it for publication.

### Conflict of Interest Statement

The author declares that the research was conducted in the absence of any commercial or financial relationships that could be construed as a potential conflict of interest.

